# Supplementary material for: Willingness to Contribute to Bio-Larviciding in the Fight against Malaria: A Contingent Valuation Study among Rice Farmers in Rwanda
Source: Int J Environ Res Public Health. 2021 Nov 4;18(21):11575. doi: 10.3390/ijerph182111575 (PMC8583195; doi:10.3390/ijerph182111575)
Supplement: Supplementary file 1 [file ijerph-18-11575-s001.zip › ijerph-1386448-supplementary.pdf]

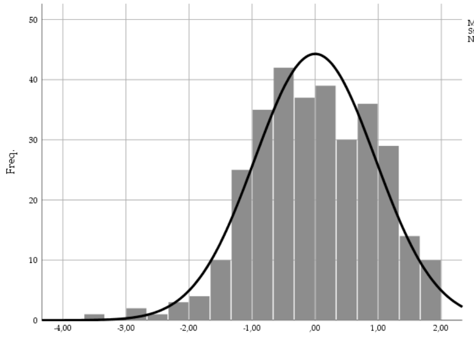

Kolmogorov-Smirnov normality test: 0.045 (p>0.05)

(a)

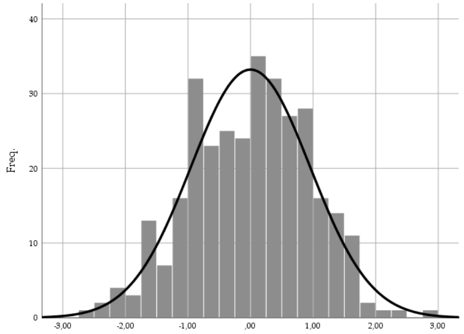

Kolmogorov-Smirnov normality test: 0.039 (p>0.05)

(b)

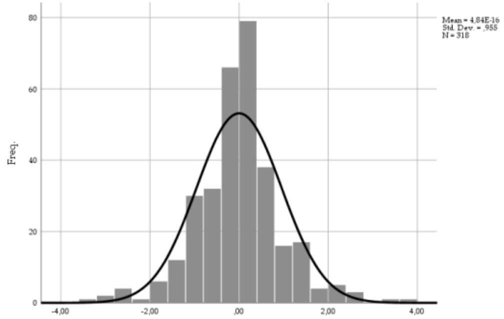

Kolmogorov-Smirnov normality test: 0.079 (p<0.05)

(c)

Figure S1. Histograms of standardized residuals from linear mixed effects model on (a) log(WTP_{LS}), (b) log(WTP_{PRO}), and (c) log(WTP_{LS} / [WTP_{PRO}*land size])

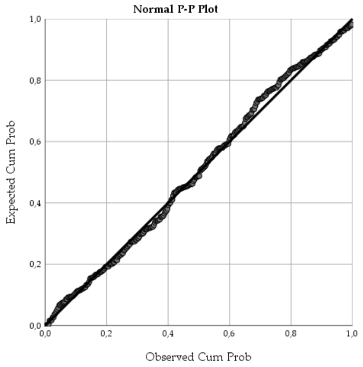

(a)

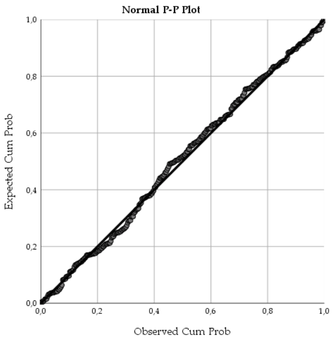

(b)

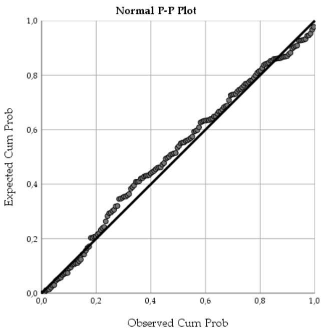

(c)

Figure S2. P-P plots of standardized residuals from linear mixed effects model on (a) log(WTP_{LS}), (b) log(WTP_{PRO}), and (c) log(WTP_{LS} / [WTP_{PRO}*land size])
